# Study of Morphology Control of Electro-Deposited Silver on Electro-Chemically Exfoliated Graphene Electrode and Its Conductivity

**DOI:** 10.3390/ma17122988

**Published:** 2024-06-18

**Authors:** Siwon Bak, Jongwon Shim

**Affiliations:** Department of Applied Chemistry, Dongduk Women’s University, Seoul 02748, Republic of Korea; tldnjs602@gmail.com

**Keywords:** graphene, exfoliation, silver, electro-deposition

## Abstract

Solution-processed graphene is beneficial for large-scale, low-cost production. However, its small lateral size, variable layer thickness, and uncontrollable oxidation level still restrict its widespread electronic application. In this study, a newly developed electrochemical exfoliation process was introduced, and a graphene-patched film electrode was fabricated by interfacial self-assembly. We were able to minimize the deterioration of graphene colloids during exfoliation by voltage and electrolyte modulation, but the patched structure of the graphene electrode still showed low conductivity with numerous inter-sheet junctions. Therefore, we determined the optimal conditions for the growth of fully networked silver structures on the multi-stacked graphene film by direct current electro-deposition, and these silver–graphene composite films showed significantly lowered graphene-colloid-patched film surface resistance.

## 1. Introduction

Graphene, despite its exceptional physical, chemical, and electrical properties, faces significant challenges in industrial application and commercialization. These challenges include difficulties in characterization, mass production, quality control, storage, and intrinsic properties, such as the absence of a band gap [1,2,3]. In the electronics industry, epitaxial growth is considered the most promising production method as it can produce single-atom-thick graphene with superconducting properties. However, this method requires large facilities, high energy costs, and overly complex transfer techniques [4]. On the other hand, solution-processed graphene can be produced through a relatively easy and inexpensive process [5]. Although it has worse electrical properties and a deteriorated structure, its low production cost and efficiency make it suitable for applications such as structural reinforcement, conductivity enhancement [6,7], heat dissipation [8], and water purification and desalination [9,10].

Solution-processed colloidal graphene generally refers to graphene oxide (GO) and reduced graphene oxide (RGO), which are produced by the chemical oxidation of natural or synthetic graphite and its reduced form. This also includes graphene flakes produced by physical exfoliation using long-term sonication, electrochemical exfoliation, and modification [11,12,13,14]. The goal of these exfoliation methods is to fabricate single-atom-thick, laterally wide graphene with a perfect sp^2^ structure. However, most exfoliation methods still have drawbacks despite numerous efforts. The lower oxygen content in graphene results in lower dispersion stability in most solvents, except for a few polar aprotic ones without stabilizers. The structural defects generated by oxidation cannot be perfectly restored even under extreme reduction conditions. Furthermore, the scalable purification and separation methods for graphene are still immature [15,16,17,18]. Based on Hummers’ method, single-atom-thick graphene oxide can be produced by a scalable batch process, but it requires many strong acids and a long purification time [19]. Exfoliation by sonication is much easier and can reduce the oxidation level of graphene, but it is tedious and generates structural defects due to its high energy [20]. When comparing the power consumption of sonication and electrochemical exfoliation methods, the power of ultrasonic devices varies widely depending on the size of the transducer, ranging from tens to hundreds of watts, with processing times from several hours to tens of hours. If the output is small, the required time increases, making the total power consumption similar. Electrochemical exfoliation equipment also varies in voltage and the required time depending on the exfoliation conditions, but generally operates at direct currents of tens of volts and less than 1A, completing the process within tens of minutes. Therefore, the energy consumed during electrochemical exfoliation is less than that during sonication, and it is eco-friendly, fast, and scalable [21,22]. However, the quality of electrochemically exfoliated graphene is a trade-off against production efficiency. A high exfoliation voltage shortens the time, but causes significant oxidation and defects, while low-voltage exfoliation minimizes the defects, but reduces the yield.

To address these issues, several modifications for electrochemical exfoliation have been developed, such as combining ultrasonication, adding surfactants [23,24], switching the electrodes [25], increasing the temperature or pressure [26], and applying an alternating current [27]. Electrolytes also play a crucial role in the exfoliation speed and graphene quality [28]. Electrochemical exfoliation can utilize various cations and anions. Most cathodic exfoliation methods using large cations can minimize oxidation damage, but these ions cannot penetrate the graphite’s inter-layer spacing, requiring additional treatments to separate the graphene flakes. In contrast, anodic exfoliation methods use smaller anions to infiltrate the graphite layers [29]. Higher voltage levels can accelerate exfoliation, but produce significant byproducts like carbon nano- and micro-particulates [30]. Low-quality graphene from anodic exfoliation is often due to early in-plane carbon network break-up before full layer expansion, potentially caused by the anions from electrolyte molecules and oxygen radicals from water electrolysis [31,32].

Electrochemically exfoliated colloidal graphene can surpass the electrical conductivity of chemically reduced graphene oxide (RGO), but its application is still limited. This is because the patched structure of small graphene flakes covering large areas has many inter-junctions that disrupt electron transport [33,34]. Efforts to increase the conductivity of graphene films through chemical functionalization, atomic doping, metal electro-deposition, and silver–nanowire hybrid composites have been made, but it is still not enough [35,36,37]. Issues such as the poor dispersibility of metallic nanomaterials and their inter-junctions need to be resolved. Therefore, we introduced a simple method to control the growth of silver nanostructures by electro-deposition on electrochemically exfoliated graphene-patched film electrodes [38].

## 2. Materials and Methods

### 2.1. Materials and Methods

Highly orientated pyrolytic graphite (HOPG, grade SPI-3) was purchased from SPI Supplies, West Chester, PA, USA. Ultrapure water was used in all experiments. Filtration was performed using Anodisc^®^ filters (47 mm/0.2 nm, Whatman^TM^, Maidstone, UK). 1-Butyl-3-methylimidazolium hydrogen sulfate (BMIM HSO_4_), platinum wire (520 mg, 1.0 mm), 95% sulfuric acid, N, N-dimethylformamide (DMF), and ethyl acetate (EA) were purchased from Sigma-Aldrich (St. Louis, MI, USA).

### 2.2. Electrochemical Exfoliation of Graphite

HOPG was cut to 10 × 5 × 0.5 mm in size and used as an anode electrode for our two-electrode cell set up, with platinum wire as a cathode [39] (Figure S1). In the first exfoliation stage, the intercalation of anion to graphite was performed in an electrochemical cell using 10~30 wt% BMIM HSO_4_ solution as electrolyte. Potentials of 2.4 V and 3.0 V were applied for 15 min to form expanded graphite. In the second exfoliation stage, the electrolyte was replaced with 0.1 M H_2_SO_4,_ and the reaction proceeded for 20 min. To compare the effect of voltage, the applied voltage was changed to 6 V, 8 V, and 10 V. The exfoliated product was washed using deionized water by vacuum filtration with an Anodisc^®^ filter and dispersed in DMF by ultrasonication in a sonic bath for 30 s. Then, this dispersion was centrifuged at 3000 rpm for 15 min to separate the thick particulates, followed by a second centrifugation at 8000 rpm for 30 min to remove the nanoparticles. The precipitate was re-dispersed in fresh DMF to prevent aggregation, and the final concentration of this graphene dispersion was approximately 0.01 mg/mL. This suspension was used for characterization and fabrication of graphene film.

### 2.3. Assembly of Graphene-Patched Film

First, 0.5 mL of graphene suspension was mixed with 30 mL of water, and more than 2 mL of EA was dropped on the surface of the aqueous mixture. The evaporation of EA onto the graphene suspension caused the interfacial assembly of graphene flakes. The assembly of graphene on the water surface proceeded under ambient conditions. Further, a graphene film floating on the aqueous phase was transferred to the 1.5 cm × 1.5 cm quartz glass plate and dried at 300 °C in air for about 1 min to remove any residual solvent [40].

### 2.4. Graphene Characterization

Field-emission transmission electron microscopy (FE-TEM, Tecnai F20 G2, Hillsboro, OR, USA) and atomic force microscopy (AFM, Multimode-8, Bruker, Billerica, MA, USA) were used to analyze the graphene thickness. The surface morphology of the graphene–silver composite films was observed by field-emission scanning electron microscopy (FE-SEM, HITACHI SU8010, Tokyo, Japan). The elemental composition (carbon and oxygen) of the exfoliated graphene samples was measured using an Organic Elemental Analyzer (OEA, Thermo Scientific FLASH 2000, Waltham, MA, USA). Raman spectroscopy (Renishaw In Via Raman Microscope, Wotton-under-Edge, UK) was used to examine the structural integrity. The sheet resistances of the graphene-assembled films and graphene–silver composite films were measured using a 4-point probe (AIT CMT-SR2000N, Suwon, Republic of Korea). Film transparency was determined using a Cintra 3030 (GBC Scientific Equipment, Keysborough, Australia, at a wavelength of 550 nm).

### 2.5. Growth of Silver Structures on Graphene Films

The interfacial assembled graphene film on a quartz plate (1.5 cm × 1.5 cm) was used as the cathode for silver structure growth. The thickness of the graphene film was controlled by the layer-by-layer (L-b-L) deposition of patched graphene film by fishing transfer from water surface. Platinum wire was used as the anode. A positive voltage (2.0~12.0 V) was applied for less than 30 min in an electrolyte of 1.0 × 10^−3^–1.0 × 10^−5^ M silver nitrate (Sigma-Aldrich, USA) in deionized water.

## 3. Results and Discussion

### 3.1. Electrochemical Exfoliation

**Figure 1:**
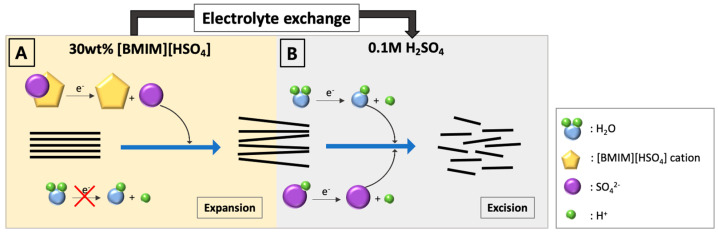
Schematic illustration of electrochemical exfoliation process.

Ionic liquids possess unique properties, such as negligible vapor pressure, thermal stability, a wide electrochemical potential window, low viscosity, good ion conductivity, and recyclability [41]. The high dielectric constant of ionic liquids provides shielding for the stacking interactions caused by van der Waals forces, which helps to disperse graphene colloids effectively. Specifically, the widened electrochemical potential window of ionic liquids can suppress the generation of hydroxyl radicals during the application of expansion voltage, thereby minimizing the oxidation of carbon layers [42,43,44]. These properties make them very useful as media for liquid/liquid extraction, electrochemistry, chemical synthesis, and catalysis [45]. In this study, we chose 1-butyl-3-methylimidazolium hydrogen sulfate (BMIM HSO_4_) as the electrolyte for the layer expanding stage. After the sulfate anions unzipped the graphite layer edges, and simultaneously the BMIM cations also expanded the electrochemical window of water (Figure 1), the onset voltage of graphite layer expansion could be slightly increased without hydrogen gas evolution by an increase in the ionic liquid amount. The stable pH near the counter electrode supported this suppressed hydrogen evolution (Table 1).

Because an increase in electrolyte solution viscosity can retard the exfoliation reaction, the ionic liquid content for the expanding stage was fixed at 30% (*v*/*v*), and we applied voltages for 15 min. However, under 2.4 V, layer expansion did not occur. Further, the expanded graphite electrode was immersed into dilute sulfuric acid (0.1 M), and the applied voltage was increased to 10 V for excision. In this condition, a higher voltage accelerated graphene layer excision. After purification by centrifugation, the exfoliated graphene was re-dispersed in *N*,*N*-dimethylformamide (DMF) or *N*-methyl-pyrrolidone (NMP). This graphene suspension initially was dark brown in color, but turned black within 24 h at room temperature due to auto-reduction (Figure S2). We presume that DMF could weaken or break the oxygen–carbon bonds, thereby detaching oxygen from graphene. This presumption is supported by several previously reported studies. For instance, graphene oxide in pure DMF could be reduced by applying microwaves without any chemical reducing agents, and the graphene oxide colloids dispersed in pure NMP were reduced by simple refluxing [46,47].

**Figure 2:**
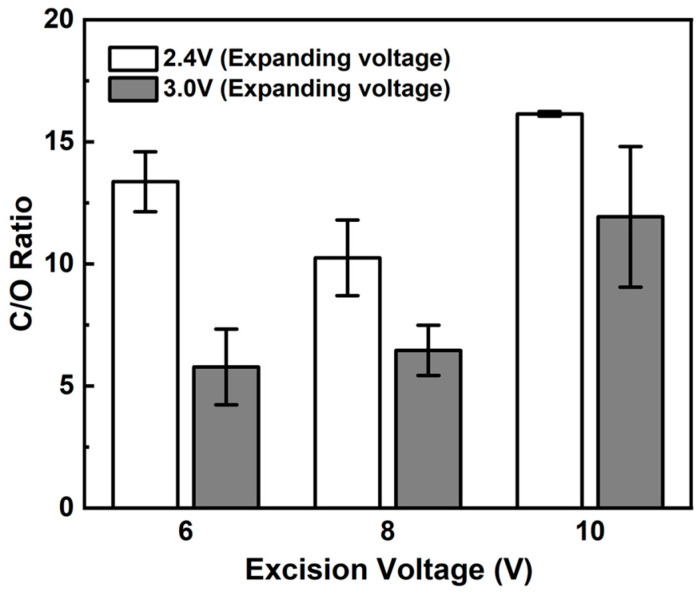
Carbon-to-oxygen ratios by atomic analysis (AA) of exfoliated graphene samples.

Figure 2 shows the graphene oxidation state in our two stage-electrochemical exfoliation process involving carbon layer expansion and excision. To determine the optimized voltage that minimizes oxidation, we adjusted the voltage during the expanding stage (2.4 V and 3 V) and the excision stage (6 V, 8 V, and 10 V), resulting in six different graphene colloids. We measure the carbon-to-oxygen (C/O) ratio of each colloid sample using elemental analysis to assess the degree of oxidation. The results showed that the C/O ratio varied between 5.7 and 16.1 depending on the conditions. An increase in voltage during the expanding stage significantly increased oxidation. However, during the excision stage, increasing the voltage from 6 V to 8 V resulted in a slight increase in oxidation, while at 10 V, oxidation significantly decreased. We presumed that the excision reaction at 10 V had a much shorter processing time than those at the other voltages, which compensated for the oxidation increase caused by voltage elevation.

**Figure 3:**
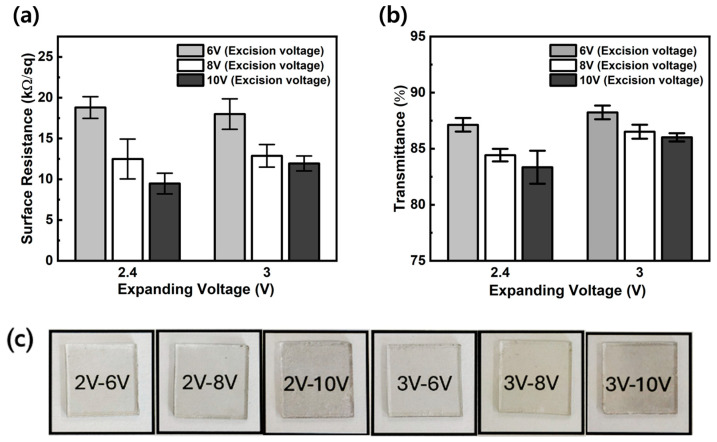
(**a**) Surface resistance of exfoliated graphene assembled film electrode on quartz plate and (**b**,**c**) transmittance.

The surface resistance and transparency of the graphene film electrodes on the quartz plate, which were assembled by interfacial self-assembly, were decreased with the increase in excision voltages. Meanwhile, the expanding voltage did not significantly affect surface resistance and transmittance lowering (Figure 3). Therefore, it was supposed that the excision voltage could increase the exfoliated graphene thickness, and it resulted in an electric conductivity increase and transmittance lowering. These results are partially similar to the findings reported by Mario Hoffmann and colleagues in 2015 regarding the effects of electrochemical exfoliation on the quality of graphene [48]. However, unlike their study, which differentiated the graphene exfoliation process into ion intercalation and layer expansion, our study combines intercalation and expansion into a single step and defines the excision of carbon layers as a second step. This approach allowed us to better understand the impact on graphene quality.

**Figure 4:**
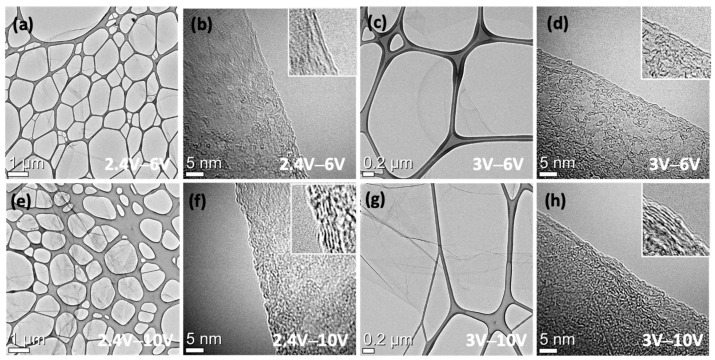
TEM images of exfoliated graphene by expanding and excision voltages. (**a**,**b**) 2.4 V of expanding and 6 V of excision. (**c**,**d**) 3 V of expanding and 6 V of excision. (**e**,**f**) 2.4 V of expanding and 10 V of excision. (**g**,**h**) 3 V of expanding and 10 V of excision.

Transmission electron microscopy (TEM) analysis showed that the number of atomic layers of this exfoliated graphene depends on the second exfoliation voltage. Most of the graphene flakes exfoliated at 6 V showed a 2–3-atom layer thickness, while those exfoliated at 10 V had 7–8 carbon layers (Figure 4).

**Figure 5:**
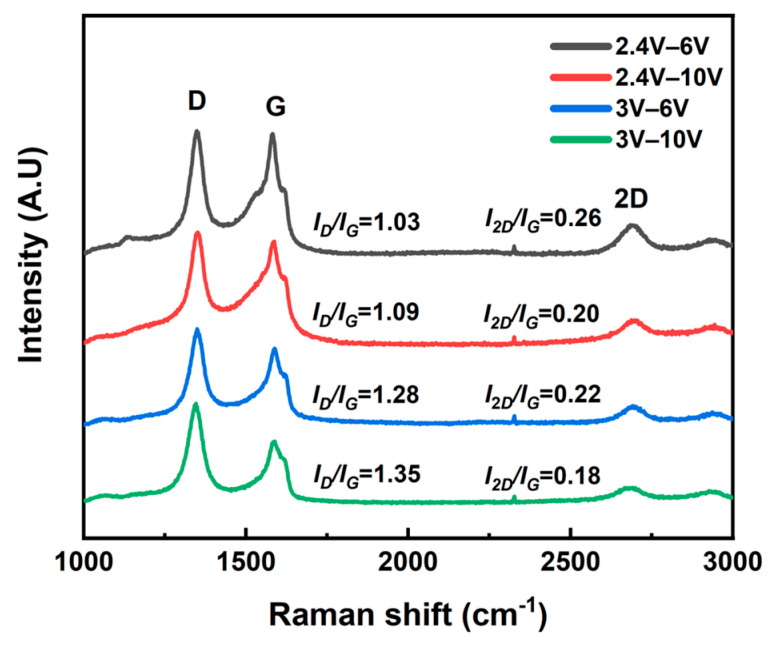
Raman spectrum of electrochemically exfoliated graphene samples with varying applied voltages.

We conducted the regression analysis of transparency and surface resistance as a function of the applied voltages during the exfoliation process. These results are shown in the Supplementary Information (Figure S3). It was indicated that the surface resistance and transmittance of the graphene-assembled films mainly depend on the excision voltage, whereas the expansion voltage affected the oxygen levels of the graphene. This was supported by Raman spectrum analysis (Figure 5). In ideal, pristine single-layer graphene, a clear G peak (1580 cm^−1^) and 2D peak (2690 cm^−1^) appear due to the highly ordered carbon structure. However, in graphene structures damaged by the exfoliation process, D and D’ peaks (1350 cm^−1^ and 1620 cm^−1^) appear. The intensity ratio of the D peak to the G peak (I_D_/I_G_) is used to express the degree of structural damage in graphene, with a high defect density indicated by a high I_D_/I_G_ value and a low defect density indicated by a low I_D_/I_G_ value [49]. Therefore, the graph in Figure 5 shows the structural damage to graphene increases with the rise in both the expanding voltage and excision voltage during exfoliation, with more significant damage caused by the expanding voltage. Additionally, as the thickness (number of layers) of exfoliated graphene increases, the width of the 2D peak becomes wider and its intensity decreases [50]. The intensity ratio of the 2D peak to the G peak (I_2D_/I_G_) was observed, and it was found that I_2D_/I_G_ decreases more significantly during the excision voltage increase (Figure S6).

Although we could control the quality of the electrochemically exfoliated graphene flakes, the lowest surface resistance of these graphene-flake-patched films was around 5~10 kΩ/sq, and the transmittance at 550 nm was 85%, and this is mainly due to the many inter-junctions of the graphene colloids [51]. We also concluded that merely improving the quality of the graphene colloids themselves using this electro-chemical exfoliation method is insufficient to mitigate this high surface resistance of large-area graphene electrodes. The performance of this graphene-patched film electrode falls short of practical levels because the typical transmittance of commercial ITO electrodes exceeds 85% at 550 nm, with a surface resistance of less than 100 ohms per square centimeter.

### 3.2. Layer Stacking and Electro-Deposition of Silver

To overcome this limitation, we attempted two-way approaches, the multi-stacking of graphene-patched films, and the hybridization of the graphene-patched film electrode with a silver nanostructure by direct electro-deposition with silver ions. It was reported that reducing silver on the graphene surface can enhance a graphene conductivity, and the growth of the silver nanostructure on the graphene surface can be controlled by the optimization of the deposition voltages, silver ion concentration, and deposition time [52,53]. Firstly, We transferred the self-assembled graphene-patched film from the air–water interface to the quartz plate and increased the number of layers from one to three. (Figure S4), and we tried to find out the optimum condition for high conducting electrode fabrication by controlling the silver nitride concentration, deposition time, and voltage. We approximately calculated the current density of the graphene film electrode. The resulting transparency and surface resistance were 93~86% and 2.4~14 KΩ/sq (Figure S5). The current density values under varying deposition conditions are summarized in Table 2 using following Equation (1):

(1) $$J=\left[\frac{V-\left(E{}^{\circ}-\left(\frac{RT}{nF}\right)\ln\left[C\right]\right)}{Rs}\right]/A$$

(*J*: current density, *A*: area of electrode, *V*: applied voltage, *Rs*: surface resistance, *E*°: standard reduction potential of silver, *n*: number of electrons transferred, *C*: silver ion concentration).

**Figure 6:**
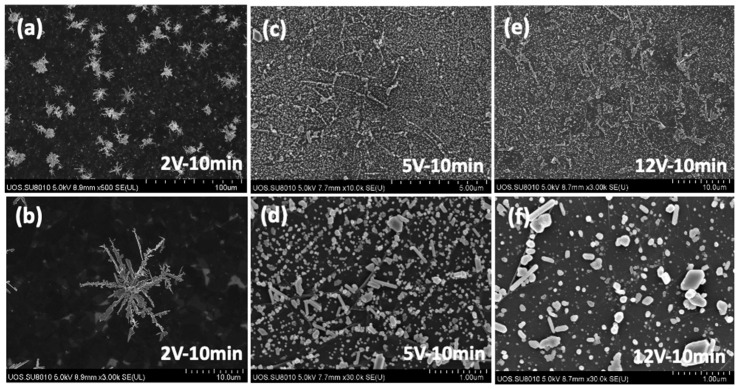
SEM imaged of electro-deposited silver on graphene-patched film electrode with varying deposition voltages (silver nitride concentration of 1.0 × 10^−4^ M).

The deposition voltages were applied from DC 2 V to 12 V for 10 min in a silver nitride concentration of 1.0 × 10^−4^ M to the patched graphene electrode having 14 KΩ/sq of surface resistance. The increased voltage led to an increase in the size, shape, and density of silver particles on the graphene film surface, but electrical conductivity did not increase significantly, and this was due to the lack of connectivity between particles (Figure 6).

**Figure 7:**
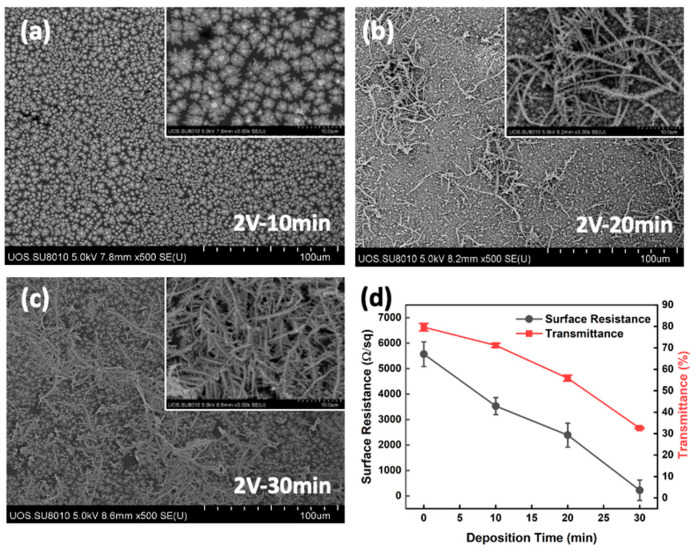
(**a**–**c**) SEM images of electro-deposited silver on double-layered graphene-patched film electrodes with 2 V deposition voltage (silver nitride concentration of 1.0 × 10^−4^ M) and (**d**) surface resistance and transmittance.

**Figure 8:**
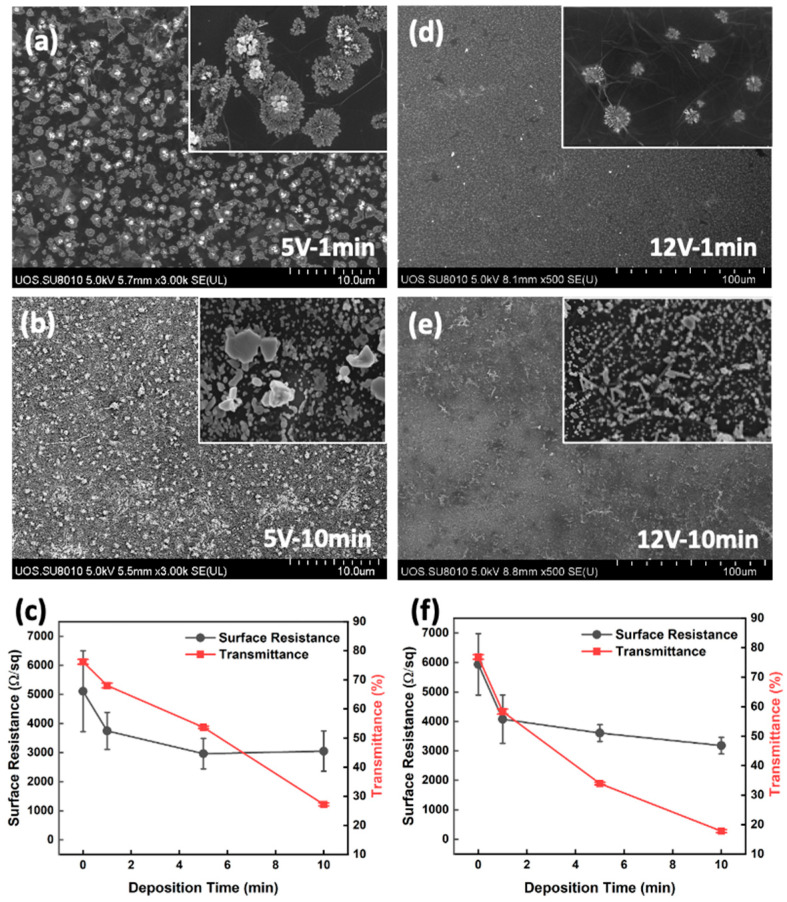
(**a**,**b**) SEM images of electro-deposited silver on double-layered graphene-patched film electrodes with 2 V deposition voltage (silver nitride concentration of 1.0 × 10^−4^ M) and (**c**) surface resistance and transmittance. (**d**,**e**) SEM images of electro-deposited silver on double-layered graphene-patched film electrodes with 5 V deposition voltage (silver nitride concentration of 1.0 × 10^−4^ M) and (**f**) surface resistance and transmittance.

Meanwhile, when the graphene film was duplicated to two layers, the surface resistance dropped below 5.5 KΩ/sq, and under these conditions, the silver structures grew dendritically and fully covered the entire electrode surfaces at 2 V. These silver dendrites were fused and entangled, resulting in a surface resistance decrease to 20 Ω/sq in 30 min. This was 200 times lower than that without silver deposition, but the 80% transmittance before deposition decreased to 32% simultaneously (Figure 7). Contrastingly, at the same silver nitride concentration, elevating the deposition voltage to 5 V and 12 V did not make the dendritic networked silver structure, and it could not lower the surface resistance significantly, but the transmittance decreased gradually (Figure 8).

Although electro-deposition on the 14 KΩ/sq surface resistance electrode and the 4.3 KΩ/sq surface resistance electrode at the same 12 V deposition voltage and ion concentration showed quite different silver morphologies; both the 12 V deposition voltage on the 14 KΩ/sq surface resistance electrode (12 V-14 K) and the 5 V deposition voltage on the 5.1 KΩ/sq surface resistance electrode (5 V-5.1 K) have similar current densities, representing identical silver morphologies. We presumed that the transformation of silver structures by direct electro-deposition could depend on the current density of the electrode, but the surface resistance of the graphene-patched film electrode more strongly affected the interconnected structure.

**Figure 9:**
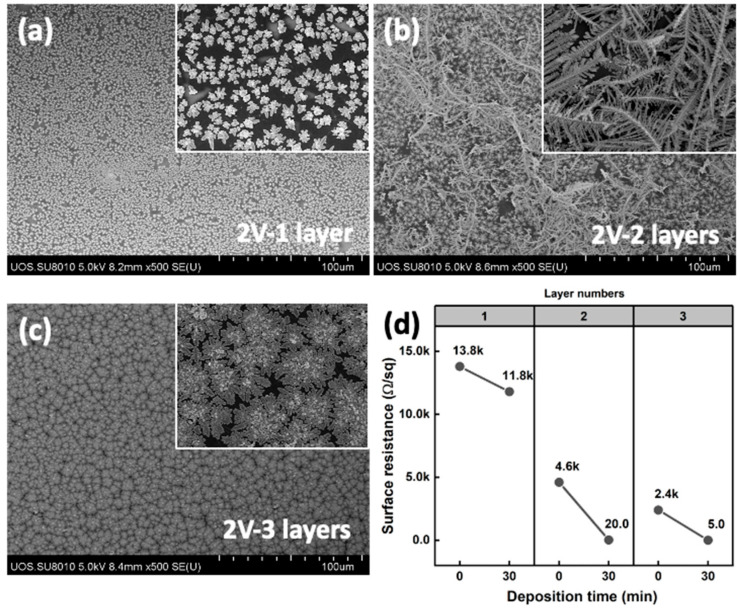
(**a**–**c**) SEM images of electro-deposited silver on single-, double-, and triple-layered graphene-patched film electrodes with 2 V deposition voltage (silver nitride concentration of 1.0 × 10^−3^ M) and (**d**) surface resistance.

Therefore, we controlled the surface resistance of the graphene electrodes using the graphene film stacking number. Under the same deposition conditions, the silver structures were not interconnected on a single-stacked graphene film, which had a surface resistance of around 13.8 KΩ/sq, but on the double-stacked graphene electrode, which showed a surface resistance of 4.6 KΩ/sq, the dendritically grown silver structures were interconnected, resulting in a surface resistance of under 20 Ω/sq. The triple-stacked graphene electrode, which has 2.4 kΩ/sq of initial surface resistance, large-sized, flattened silver structures widely spread out, and its surface resistance went down to below 5 Ω/sq (Figure 9). Because we controlled the surface resistance of these graphene electrodes by film layer stacking, we presumed that these interconnected silver structures could not be determined by the conductivity enhancement of the electrode, but the surface roughness and homogeneity of the graphene-patched film.

Additionally, we performed the regression analysis of surface resistance considering multiple variables, including the staking number of graphene-patched films (Figure S6). We determined that the deposition voltage and silver ion concentration did not significantly affect the surface resistance of the silver–graphene composite film electrodes. However, only the staking number of the graphene-patched films can create fully connected silver structures and significantly reduce the surface resistance.

## 4. Conclusions

We successfully controlled the quality of the graphene colloids using a modified electrochemical exfoliation method. By switching the electrolyte composition and adjusting the voltage, we optimized the expansion and excision reactions. The optimized ionic liquid significantly reduced the electrolysis of water molecules, suppressing oxygen radical generation during the expanding reaction. Additionally, a low working voltage and short reaction time in the excision reaction minimized the deterioration of the graphene quality. Utilizing this high-quality solution-processed colloidal graphene, we fabricated self-assembled graphene films. These films demonstrated a low surface resistance of around 5~15 KΩ/sq and 85% transmittance at 550 nm, although they were not comparable to commercial ITO electrodes. Therefore, we prepared a silver–graphene film composite, transforming the morphology of electro-deposited silver for high conductivity. On double-stacked graphene film electrodes, which have a surface resistance under 5 KΩ and around 80% transmittance at 550 nm, the silver structures were fully interconnected, significantly lowering the junction resistance between the graphene colloids.

## 5. Future Works

Through this study, we identified that multi-stacking graphene-patched films are necessary to grow fully connected silver structures on a patch structure connected by small graphene colloids. However, we were unable to determine the specific effects of this film stacking. We speculate that this issue is due to the resistance lowering at the overlapping regions of graphene colloids and the numerous empty spaces in the patch structure, leading to an uneven current density distribution across the assembled film. This, in turn, prevents the effective reduction of silver ions in these areas. To investigate this hypothesis, future research will involve high-resolution current density analysis and electromagnetic field distribution studies. Additionally, silver nanoparticle-decorated graphene composite film electrodes have been reported as surface-enhanced Raman scattering (SERS)-active substrates [54,55]. We plan to evaluate and optimize the sensitivity of these signals according to various silver structures and explore their potential development as sensors for detecting different functional molecules.

## Figures and Tables

**Table 1:** Expansion onset voltage.

Ionic Liquid Concentrations (%)	Initial pH	Applied Voltage (V)	Final pH
10	1.3	1.9	1.26
20	1.18	2.1	1.25
30	1.08	2.4	1.19
40	1.04	2.7	1.05
50	1.01	3	1.03

**Table 2:** Deposition conditions and calculated current density.

Ion Concentration (M)	Deposition Voltage (V)	Electrode Area (cm^2^)	Surface Resistance (KΩ/sq)	Current Density (mA/cm^2^)
1 × 10^−4^	2	2.25 (1 L)	14	0.31
1 × 10^−4^	5	2.25 (1 L)	14	1.26
1 × 10^−4^	12	2.25 (1 L)	14	3.48
1 × 10^−4^	2	2.25 (2 L)	5.5	0.78
1 × 10^−4^	5	2.25 (2 L)	5.1	3.45
1 × 10^−4^	12	2.25 (2 L)	4.3	11.33
1 × 10^−3^	2	2.25 (1 L)	13.8	0.33
1 × 10^−3^	2	2.25 (2 L)	4.6	0.99
1 × 10^−3^	2	2.25 (3 L)	2.6	1.75

## Data Availability

The original contributions presented in the study are included in the article and Supplementary Material, further inquiries can be directed to the corresponding author.

## Supplementary Materials

The following supporting information can be downloaded at: https://www.mdpi.com/article/10.3390/ma17122988/s1, Figure S1: (a) Schematic illustrations of conventional electrochemical exfoliation and (b) electrolyte-switching electrochemical exfoliation. (c) Multi-layer stacking of self-assembled graphene film; Figure S2: (a) HOPG(anode) and platinum wire(cathode) electrode before applying voltage. (b) After layer expansion in 15 min 2.4 V DC, electrolytes solution exchanged. (c) Graphene layer excision under 6 V DC in diluted sulfuric acid. (d) Dark brown color of exfoliated graphene colloids in DMF. (e) Reduced graphene colloids after 24 h (f) Color comparison of diluted graphene colloids in DMF; Figure S3: Linear regression analysis of graphene patched film electrodes surface resistances by (a) expanding and (b) excision voltages (n = 47, *p* < 0.05). Linear regression analysis of graphene patched film electrodes transmittance by (a) expanding and (d) excision voltages (n = 18, *p* < 0.05); Figure S4: (a) Transmittance and surface resistance of interracial self-assembled graphene patched film transferred on quartz plate (1.5 cm × 1.5 cm). (b) Double-stacked graphene pathed film on quartz plate. (c) Triple-stacked graphene patched film; Figure S5: (a) Double-stacked graphene patched film transferred on quartz plate (1.5 cm × 1.5 cm). (b) Silver deposited (left half: 0 min, right half: 30 min) on graphene pathed film. (c) Silver deposited (left half: 20 min, right half: 30 min) on graphene pathed film; Figure S6: (a) Correlation of Raman spectrum peak intensity ratios (I_D_/I_G_ and I_2D_/I_G_) with surface resistance of graphene patched film electrodes. (b) Correlation between transmittance and surface resistance of graphene patched film electrodes; Figure S7: Linear regression analysis of silver-deposited graphene patched film electrodes surface resistances by (a) deposition voltage, (b) silver ion concentration and (c) multiplied graphene patched films (*p* < 0.05). (d) Linear regression modeling of silver-deposited graphene patched film electrodes surface resistances by variables. (*p* < 0.1).
