# Supplementary material for: Study of Morphology Control of Electro-Deposited Silver on Electro-Chemically Exfoliated Graphene Electrode and Its Conductivity
Source: Materials (Basel). 2024 Jun 18;17(12):2988. doi: 10.3390/ma17122988 (PMC11206019; doi:10.3390/ma17122988)
Supplement: Supplementary file 1 [file materials-17-02988-s001.zip › materials-3040459-supplementary.pdf]

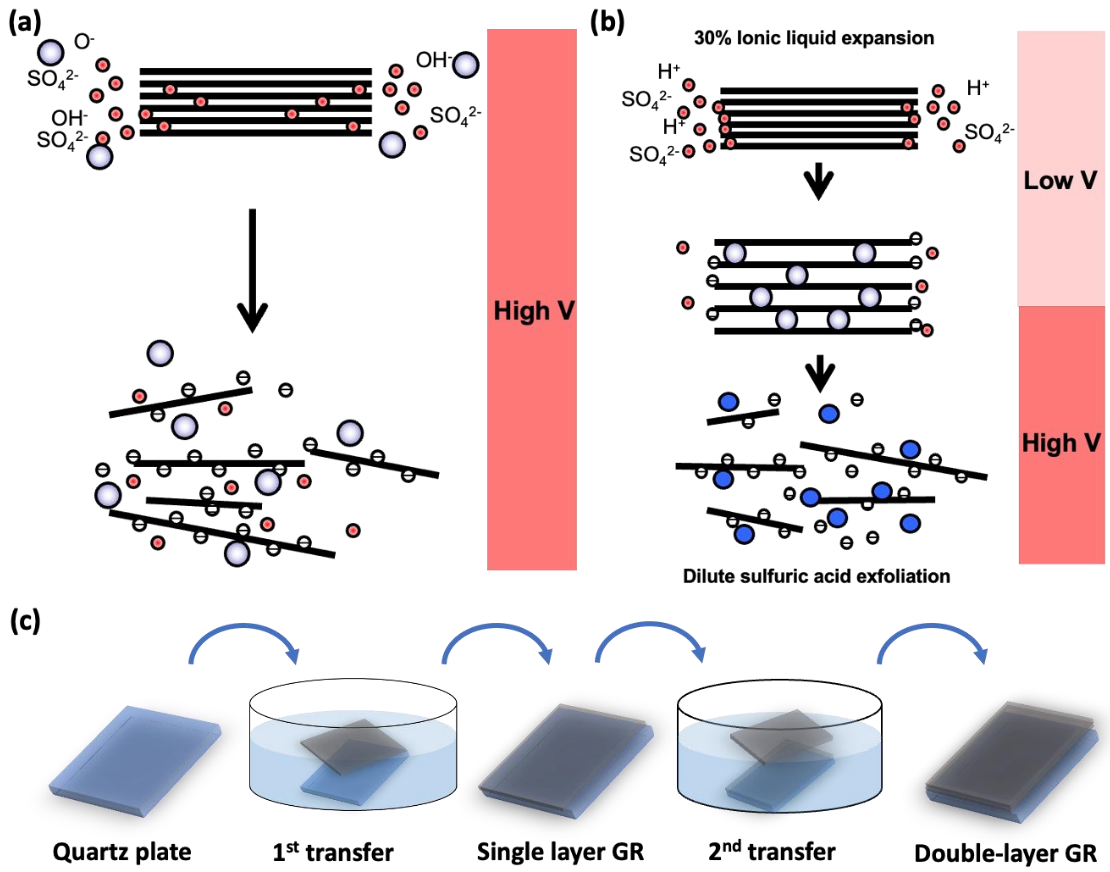

Figure S1. (a)Schematic illustrations of conventional electrochemical exfoliation and (b) electrolyte-switching electrochemical exfoliation. (c) Multi-layer stacking of self-assembled graphene film.

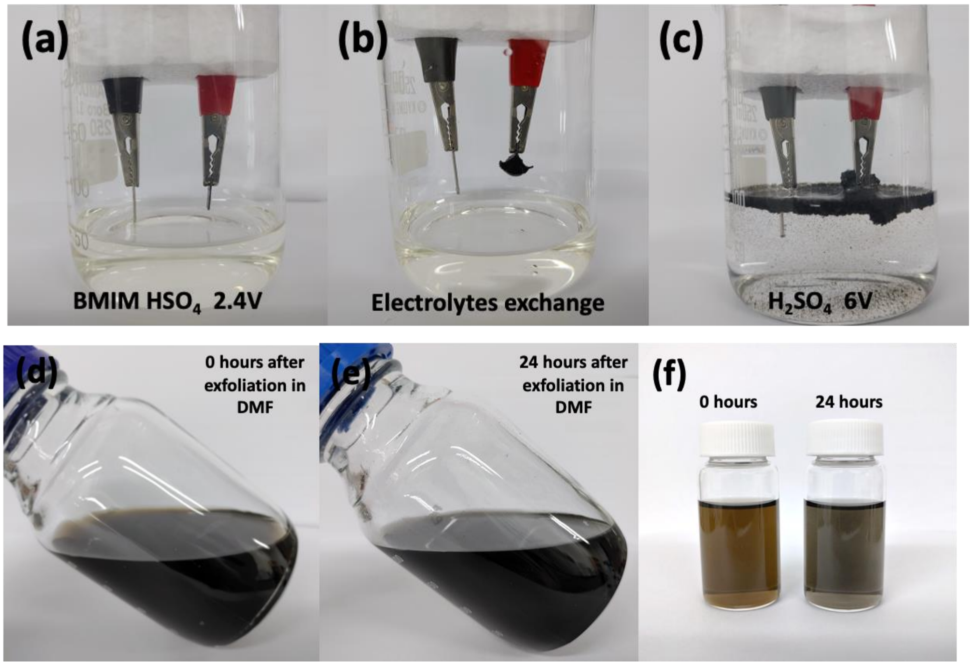

Figure S2. (a) HOPG(anode) and platinum wire(cathode) electrode before applying voltage. (b) After layer expansion in 15 minutes 2.4V DC, electrolytes solution exchanged. (c) Graphene layer excision under 6V DC in diluted sulfuric acid. (d) Dark brown color of exfoliated graphene colloids in DMF. (e) Reduced graphene colloids after 24 hours (f) Color comparison of diluted graphene colloids in DMF.

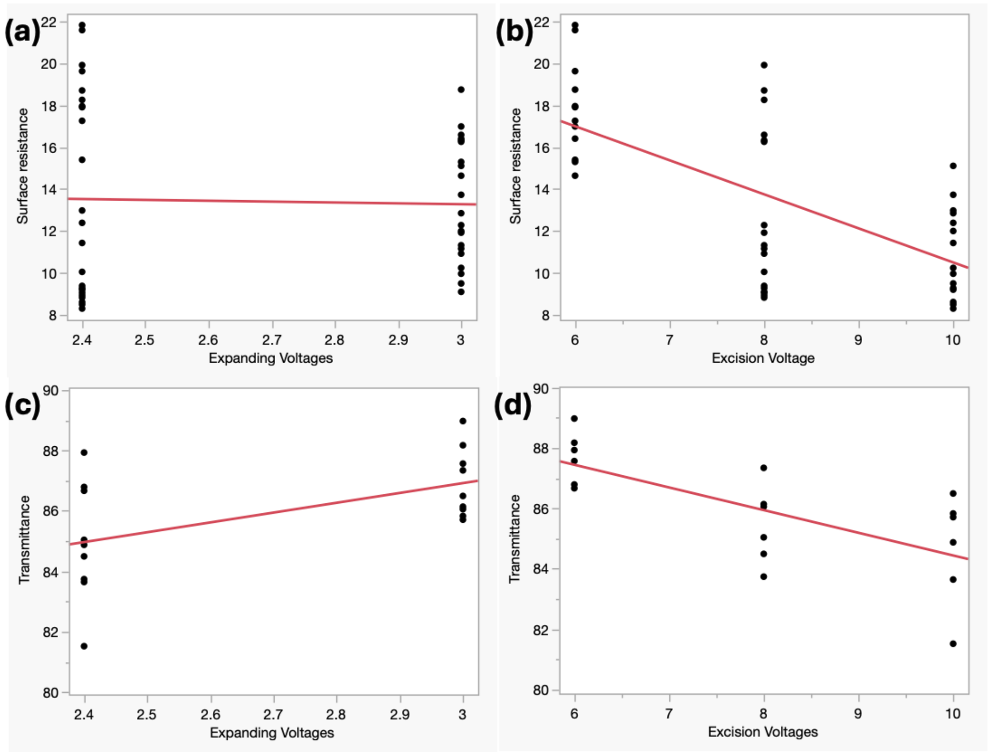

Figure S3. Linear regression analysis of graphene patched film electrodes surface

resistances by (a) expanding and (b) excision voltages ($n=47$, $p<0.05$). Linear regression analysis of graphene patched film electrodes transmittance by (a) expanding and (d) excision voltages ($n=18$, $p<0.05$)

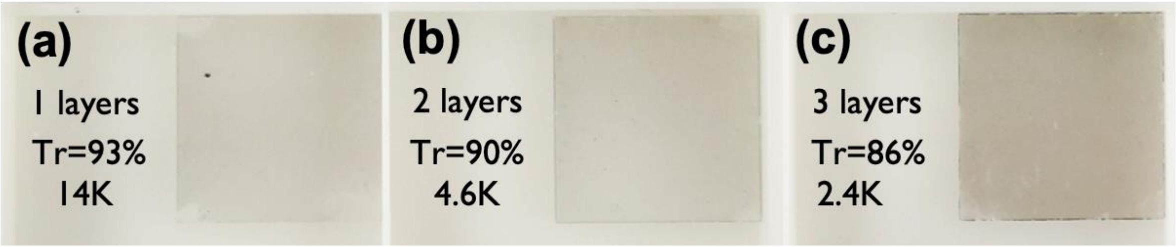

Figure S4. (a) Transmittance and surface resistance of interracial self-assembled graphene patched film transferred on quartz plate (1.5cm x 1.5cm). (b) Double-stacked graphene pathed film on quartz plate. (c) Triple-stacked graphene patched film.

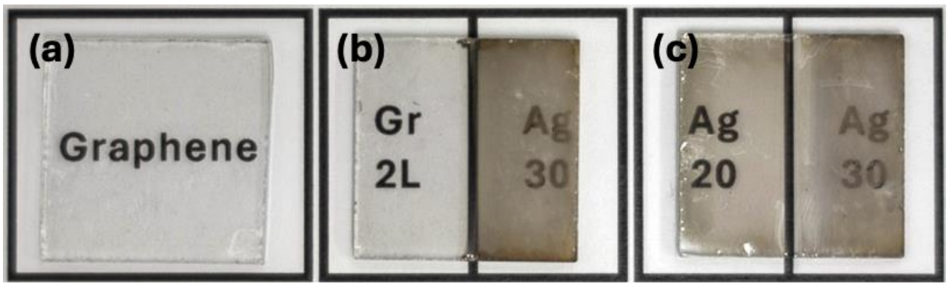

Figure S5. (a) Double-stacked graphene patched film transferred on quartz plate (1.5cm x 1.5cm). (b) Silver deposited (left half: 0min, right half: 30min) on graphene pathed film. (c) Silver deposited (left half: 20min, right half: 30min) on graphene pathed film.

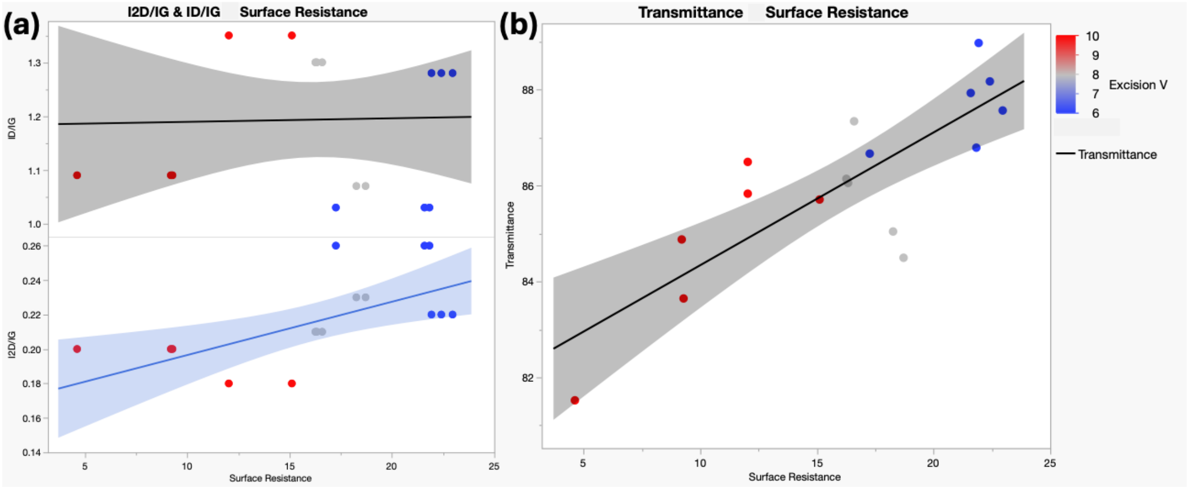

Figure S6. (a) Correlation of Raman spectrum peak intensity ratios (I_D/I_G and I_{2D}/I_G) with

surface resistance of graphene patched film electrodes. (b) Correlation between transmittance and surface resistance of graphene patched film electrodes.

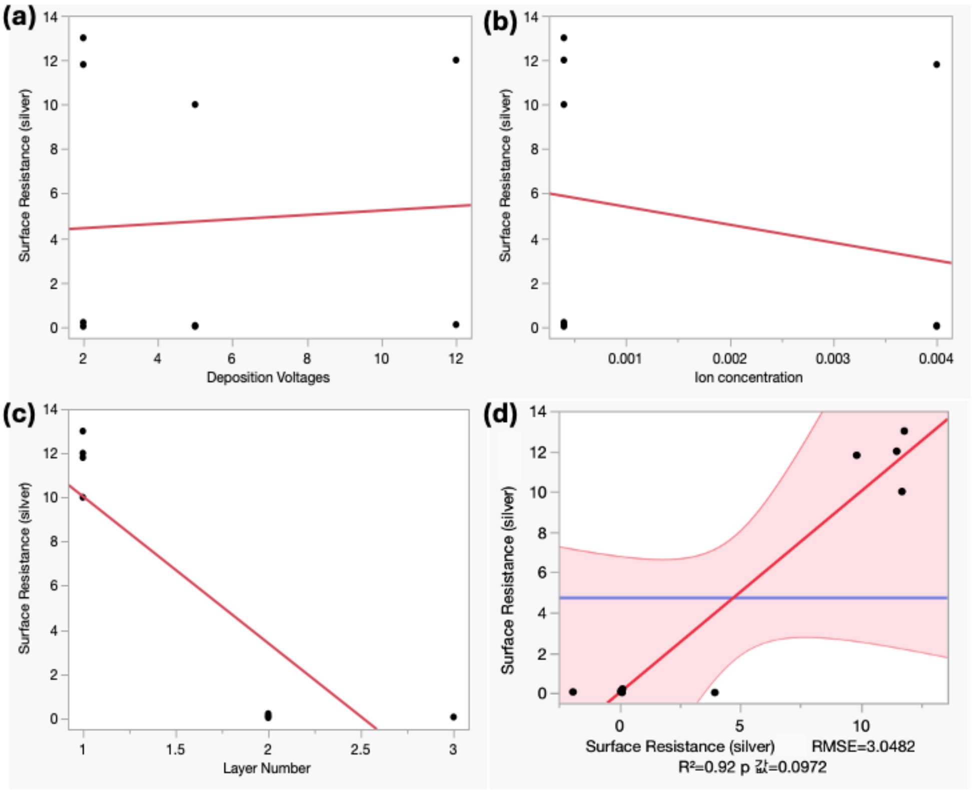

Figure S7. Linear regression analysis of silver-deposited graphene patched film electrodes surface resistances by (a) deposition voltage, (b) silver ion concentration and (c) multiplied graphene patched films ($p<0.05$). (d) Linear regression modeling of silver-deposited graphene patched film electrodes surface resistances by variables. ($p<0.1$)
